# SNHG12 promotes carcinogenesis of human renal cell cancer via functioning as a competing endogenous RNA and sponging miR‐30a‐3p

**DOI:** 10.1111/jcmm.16417

**Published:** 2021-03-30

**Authors:** Hongyuan Yu, Junlong Liu, Zhe Zhang, Yuyan Zhu, Jianbin Bi, Chuize Kong

**Affiliations:** ^1^ Department of Urology First Hospital of China Medical University Shenyang China

**Keywords:** competing endogenous RNA, miR‐30a‐3p, renal cell carcinoma, small nucleolar RNA host gene 12

## Abstract

Small nucleolar RNA host gene 12 (SNHG12) has been indicated in the tumorigenesis of various human cancers, including clear cell renal cell carcinoma (ccRCC). However, the underlying mechanisms of SNHG12 driving progression of ccRCC remain incompletely understood. In the present study, we discovered that SNHG12 is up‐regulated in ccRCC and that overexpression of SNHG12 predicted poor clinical outcome of ccRCC patients. SNHG12 knockdown notably inhibited proliferation and migration of RCC cells. Furthermore, we discovered that miR‐30a‐3p, a putative ccRCC inhibitor, was competitively sponged by SNHG12. Via the crosstalk network, SNHG12 was capable of up‐regulating multiple target genes of miR‐30a‐3p, namely, RUNX2, WNT2 and IGF‐1R, which have been identified to facilitate tumorigenesis of ccRCC. Taken together, our present study suggested a novel ceRNA network, in which SNHG12 could promote the malignancy of ccRCC although competitively binding with miR‐30a‐3p and consequently release the expression of its downstream cancer‐related genes.

## 1 INTRODUCTION

Renal cell carcinoma (RCC) accounts for 2% of the global cancer burden, and its incidence rates have been increasing.^1^ Worldwide, 403 000 new cases and 175 000 deaths as a result of RCC occurred in 2018.^2^ There are multiple histological subtypes of RCC, each characterized by a unique molecular landscape. Among them, clear cell renal cell carcinoma (ccRCC), which arises from the proximal tubule cells of the kidney nephron, is the most common subtype and accounts for 75% of all cases.^3^ Although most detected renal lesions are small and local tumours, 17% of the patients harbouring distance metastasis when diagnosed.^3^, ^4^ Despite advanced management strategies such as chemotherapy have been developed, RCC remain one of the most lethal urological malignances, and <10% of patients with metastasis are alive 5 years after diagnosed.^1^

Accumulating evidence have demonstrated that long non‐coding RNAs (lncRNAs) can function in a wide variety of biological processes and can play crucial roles in driving tumorigenesis or inhibiting tumour progression.^5^, ^6^, ^7^ Small nucleolar RNA host gene 12 (SNHG12), also acknowledged as LNC04080, is such a lncRNA and has been identified as a potential therapeutic target and a diagnostic biomarker for human cancer, including RCC.^8^, ^9^ SNHG12 is 1,867 bases long and locates at the p35.3 region on chromosome 1.^10^ It encodes a family of small nucleolar RNAs which includes 4 members, namely SNORA66, SNORA61, SNORA16A and SNORD99.^11^ Elevated expression of SNHG12 has been identified to correlate with proliferation, invasion and metastasis of human cancer cells, thereby modulating the survival of cancer patients.^12^, ^13^, ^14^

MicroRNAs (miRNAs) are another class of conserved and small non‐coding RNAs which have been identified to function in a variety of biological processes, including tumorigenesis.^15^, ^16^ The major mechanism of miRNAs facilitating cancer progression is through down‐regulating protein expression by binding to seed sequences present in the target genes and influence their biological functions.^16^ Studies profiling miRNAs have noted the dysregulation of many miRNAs in ccRCC, for example, miR‐215,^17^ miR‐200,^18^ miR‐708,^19^ miR‐199a^20^ and miR‐30a, which have been reported to regulate growth, invasion and migration of ccRCC cells. Among them, miR‐30a has been proved to function as a putative tumour suppressor in ccRCC.^21^ Studies have revealed that miR‐30a‐5p inhibits ccRCC aggressiveness through repression of zinc finger E‐box binding homeobox 2 (ZEB2)^22^ and GRP78,^23^ and miR‐30a‐3p has also been reported to suppress cellular invasion and metastasis of ccRCC via targeting autophagy related 12 (ATG12)^24^ and Wnt family member 2 (WNT2).^25^ Furthermore, a late study validated that miR‐30a‐5p can be a novel diagnostic and prognostic biomarker for ccRCC.^26^

In the present study, we have identified a novel mechanism of SNHG12 promoting the tumorigenesis of ccRCC. SNHG12 functions as a competing endogenous RNA and interacts with miR‐30a‐5p, therefore releases the expression of a series of target oncogenes and further promotes the aggressive of ccRCC cells. Our data also suggested that knockdown of SNHG12 in ccRCC cells significantly repressed in vivo tumour growth, whereas knockdown of miR‐30a‐3p partially rescued the in vivo tumour growth. This study has provided a new explanation of the mechanism through which SNHG12 functions as a oncogene and promotes the carcinogenesis of ccRCC.

## 2 MATERIALS AND METHODS

### 2.1 Tissues

For the use of clinical materials for research purposes, prior patients’ written consent and approval were obtained from the China Medical University and The First Affiliated Hospital of China Medical University. The clinical and pathological information of 90 patients with ccRCC and underwent partial cystectomies or radical cystectomies from 2018 to 2019 at the Department of Urology of the First Affiliated Hospital of China Medical University in China are collected as listed in Table 1. The tissue specimens were collected and immediately frozen in liquid nitrogen and stored at −80°C. Histologically, the tumours were classified according to the 2004 World Health Organization histological classification of RCC tumours and were staged using the 2002 American Joint Committee on Cancer system.

**TABLE 1:** The clinicopathological characteristics in 90 patients with ccRCC

Parameters	Number of cases	SNHG12		*P* value
		High	Low	
Total cases	90	45	45	
Gender				
Male	58	31	27	.3784
Female	32	14	18	
Age				
>60	31	20	11	.0559
≤60	59	25	34	
T stage				
T1	76	39	37	.3359
T2	9	3	6	
T3	3	1	2	
T4	2	2	0	
Nuclear classification				
I	5	3	2	.0302
II	59	23	36	
III	20	14	6	
IV	6	5	1	
Distant metastasis	2	1	1	–
Lymphatic invasion	0	0	0	–

### 2.2 Cell culture

The human ccRCC cell lines, OSRC‐2 and 769‐P cells were purchased from the cell bank of Chinese Academy of Sciences. The cells are maintained in RPMI 1640 (HyClone) supplemented with 10% FBS (HyClone) and 1% penicillin‐streptomycin (HyClone) at 37°C and regularly tested to ensure that they are mycoplasma‐free.

### 2.3 RNA isolation and real‐time quantitative PCR

Total RNA from cultured cells and fresh surgical RCC or normal renal tissues was extracted using a miRNeasy™ Mini Kit (Qiagen), according to the manufacturer's instructions. cDNA synthesis and quantitative real‐time PCR were performed with a mercury LNA™ Universal RT microRNA PCR kit (Exiqon, Skelstedet). The hsa‐miR‐30a‐3p and U6 LNA™ PCR primer sets were purchased from Exiqon. qRT‐PCR was performed with SYBR^®^ Premix Ex Taq™ (Tli RNaseH Plus; Takara Biotechnology CO. LTD.) and LightCycler™ 480 II system (Roche). β‐actin and U6 snRNA were used as endogenous controls for mRNA and miRNA, respectively. The primers used to amplify SNHG12 is: forward: TCTGGTGATCGAGGACTTCC, reverse: ACCTCCTCAGTATCACACACT. The relative levels of expression were quantified and analysed using LightCycler™ 480 software 1.5.1.6.2 (Roche). The 2^−ΔΔCT^ method was performed to calculate the relative expression, and expression levels of negative controls were used for calibration. Three independent experiments were performed to analyse the relative gene expression.

### 2.4 Plasmids and transfection

The luciferase reporter plasmids were constructed by GenePharma. shRNAs were constructed using pLKO.1 vector according to Addgene TRC Cloning Protocol. The microRNA agomir and antagomir were purchased from GenePharma. Transfections were performed with the Lipofectamine 3000 Reagent (Invitrogen) following the manufacturer's protocol. Final concentrations for miRNA agomir/antagomir or plasmids were 50 nmol/L and 0.75 μg/mL. Cells were cultured in a six‐well plate with 2 mL culture medium. The concentration for lentivirus transduction was 5 × 10^6^ transducing units of lentivirus. The stable cell lines were constructed using puromycin (200 μg/mL).

### 2.5 Luciferase reporter assay

Luciferase reporter assay was performed with a Dual Luciferase Reporter Assay Kit (Promega) according to the manufacturer's protocol. The luciferase reporters, wild/mutant type of psiCHECK2‐SNHG12 and wild/mutant type of psiCHECK2‐RUNX2 were purchased from GenePharma. The luciferase reporter construct was co‐transfected with agomir of miR‐30a‐3p into ccRCC cells by Lipofectamine 3000 (Invitrogen) according to the manufacturer's guidelines. The relative luciferase activity was measured by Synergy HTX multi‐mode microplate reader (BioTek).

### 2.6 Western blotting analysis

Antibodies to RUNX2 (1:1000, #12556, CST), WNT2 (1:1000, ab109222, Abcam), IGF‐1R (1:1000, #3027, CST) and GAPDH (1:1000, #5174, CST) were used according to the manufacturers’ protocols. The Western blotting analysis was performed as described before. Briefly, equal amounts of protein extracts were separated by 10% SDS‐polyacrylamide gel electrophoresis (SDS‐PAGE) and transferred to polyvinylidene fluoride (PVDF) membranes (Millipore). The membranes were blocked with Tris‐buffered saline plus Tween‐20 (TBS‐T; 0.1% Tween‐20) with 5% (w/v) non‐fat dry milk and were then incubated with primary antibodies in TBS‐T at 4°C overnight. After three washes with TBS‐T for 15 minutes each, the membranes were incubated with the appropriate HRP‐labelled secondary antibodies for 1 hour at 37°C. The immunobands were visualized using the ECL reagents (Transgen Biotechnology) on a MicroChemi Chemiluminescent Imaging System (DNR Bio‐Imaging Systems, Mahale HaHamisha). The densitometric values for each band were calculated by Image J 1.46r software (Wayne Rasband, National institutes of Health), and the statistical analysis was conducted based on the ratios of target protein/GAPDH.

### 2.7 5‐Ethynyl‐2′‐deoxyuridine (EdU) assay

The cells were planted in 24‐well plates. After 48 hours transfection, EDU (1:1000) reagent was added (BeyoClick™, EDU‐488). Culture was continued for another 2 hours, and experiments were conducted according to the manufacturer's instructions. Finally, the number of proliferating cells was counted by taking photographs under a fluorescence microscope (Olympus Corporation).

### 2.8 Transwell assay

The transwell assay was performed to evaluated cell invasive capacities using the transwell chamber (Corning) without matrigel (BD Biosciences) according to the manufacturer's instructions. Cells were re‐suspended in RPMI 1640 containing 1% FBS, and 0.2 mL cell suspension (1 × 10^4^/mL) was seeded into the top chamber, whereas 0.6 mL of RPMI 1640 containing 10% FBS was filled in the lower chamber as the chemoattractant. After 24 hours of incubation at 37°C with 5% CO_2_, the number of cells invaded to the lower chamber was counted in 3 randomly selected visual fields, and the images were captured by a Leica DM3000 microscope (Leica).

### 2.9 Immunohistochemistry

The expression of ki67 was detected using an UltraSensitive™ SP (Mouse/Rabbit) IHC kit (Maxin‐Bio) according to the manufacturer's instructions. Briefly, sections were firstly dewaxed in xylene and ethanol, and antigen retrieval was performed with a microwave for 10 minutes at 100°C. The sections were then incubated with antibodies for 1 hour, followed by biotinylated anti‐IgG antibody and streptavidin‐biotinylated complex horseradish peroxidase. DAB and haematoxylin were used for nuclear staining. The images were then captured by upright metallurgical microscope (Olympus) under an original magnification of 200×. Pathological diagnosis and analysis in this study were performed by two independent pathological specialists.

### 2.10 Animal experiments

BALB/c nude mice (4‐6 weeks old, 14‐16 g) were purchased from Beijing Vital River Experimental Animal Technology Co. Ltd., and housed in Department of laboratory animal science of China Medical University. The study was approved by Medical Laboratory Animal Welfare and Ethics Committee of China Medical University, and the methods were carried out in accordance with the approved guidelines. The engineered OSRC‐2 cells were separately injected into the flanks of athymic nude mice to establish a xenograft tumour. When tumour sizes reached approximately 20 mm^3^ (approximately 1 week after inoculation), the mice were randomly divided into 3 groups (one group was established by NC‐OSRC‐2 cells and the other two by pLK‐SNHG12‐OSRC‐2 cells; 6 mice/group), and the antagomir of miR‐30a‐3p (diluted in PBS at 2 μmol/L) or 100 μL of negative control was injected intratumourally twice per week in each group for 5 weeks. Tumours were examined twice weekly; the length and width were measured with callipers, and tumour volumes were calculated using the equation (Length × Width^2^)/2. Forty‐five days after injection, the animals were killed, and the tumours were excised, weighed, paraffin‐embedded and subjected to staining assays.

### 2.11 RNA immunoprecipitation

769‐O cells were lysed by TRIzol reagent (Invitrogen), and total RNAs including miRNA were extracted and prepared for further test. The recombinant specific SNHG12 binding probes or mutated probes are synthesized by GenePharma. 1 μg of each RNA sample was incubated without/with empty probe or with 5 μg of biotin‐labelled MREs mutated SNHG12 anti‐sense probe or biotin‐labelled SNHG12 anti‐sense probe overnight at 4°C, and the complexes were isolated with streptavidin agarose beads (Invitrogen). Expression of miR‐30a‐3p was measured using real‐time PCR analysis.

### 2.12 Statistical analysis

Data are shown as mean ± SEM from at least three independent experiments. Statistical analyses involved Student's *t* test, one‐way ANOVA, Fisher's exact test and Kaplan‐Meier analysis with SPSS 22 (IBM Corp.) or GraphPad Prism 6 (GraphPad Software, Inc). Pearson's correlation coefficient analysis was used to determine the correlation of expression between the genes. *P* < .05 was considered statistically significant.

## 3 RESULTS

### 3.1 Expression of SNHG12 is up‐regulated in ccRCC tissues, and high expression of SNHG12 is closely associated with poor prognosis of patients with ccRCC

**FIGURE 1:**
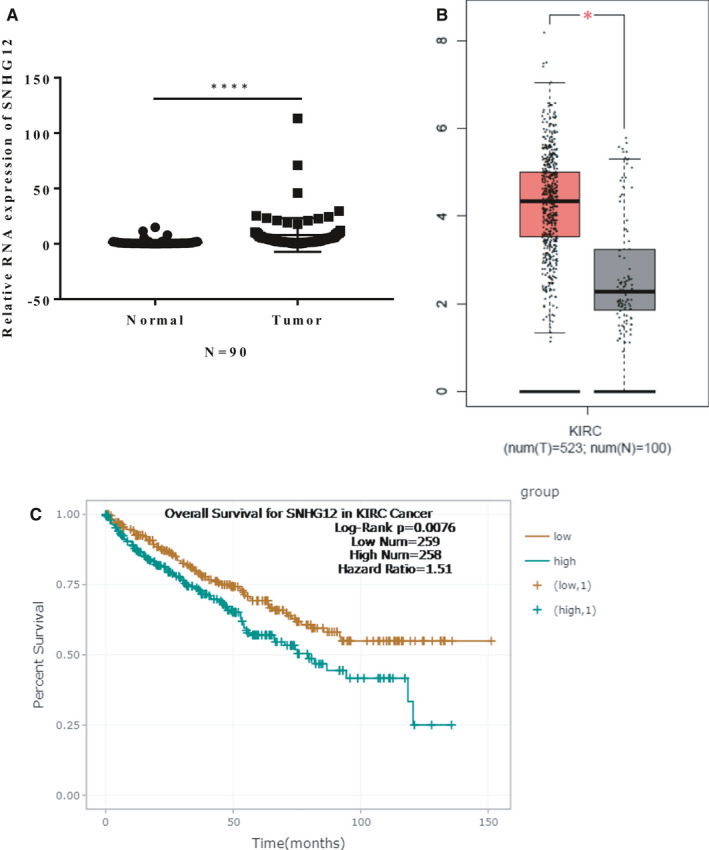
SNHG12 is up‐regulated in ccRCC tumour tissues, and high expression of SNHG12 is closely associated with poor overall survival of patients with ccRCC. A, SNHG12 is significantly up‐regulated in ccRCC tumour tissues compared with adjacent normal tissues. (n = 90, *P* < .001). B, Data from TCGA database suggested that expression of SNHG12 in ccRCC tumour tissues (n = 523) is much higher than that in normal kidney tissues (n = 100, *P* < .001). C, Overall survival for patients with ccRCC were analysed using Kaplan‐Meier analysis, and the patients were divided into two groups based on SNHG12 expression (High SHNG12 expression: 258; Low SHNG12 expression: 259). The survival data were also from TCGA database and analysed by ENCORI software. Results suggested that high expression of SNHG12 is closely associated with poor overall survival of ccRCC patients. (*P* = .0076, hazard ratio = 1.51)

It has been acknowledged that SNHG12 is dysregulated in many cancers. First, we measured the expression of SNHG12 in 90 pairs of ccRCC tumour samples and adjacent normal renal tissue samples by qRT‐PCR. The clinicopathological features of the patients are listed in Table 1. Our data showed that expression of SNHG12 is significantly up‐regulated in ccRCC tumour samples (Figure 1A). Next, we analysed the expression of SNHG12 in ccRCC tumour tissues based on the data from The Cancer Genome Atlas (TCGA) database using ENCORI software.^27^ The results were consistent with our findings that SNHG12 is significantly up‐regulated in ccRCC tumours (n = 523) compared with normal renal tissues (n = 100) (Figure 1B). Moreover, high expression of SNHG12 is significantly correlated with poor overall survival of ccRCC patients (Figure 1C). Taken together, above data suggested that expression of SNHG12 is significantly elevated in ccRCC tumour tissues, and high expression of SNHG12 is positively correlated with poor survival of ccRCC patients. These results revealed that SHNG12 could be a potential therapeutic target and prognostic marker for ccRCC patients.

### 3.2 Knockdown of SNHG12 represses proliferation and migration of ccRCC cells in vitro

**FIGURE 2:**
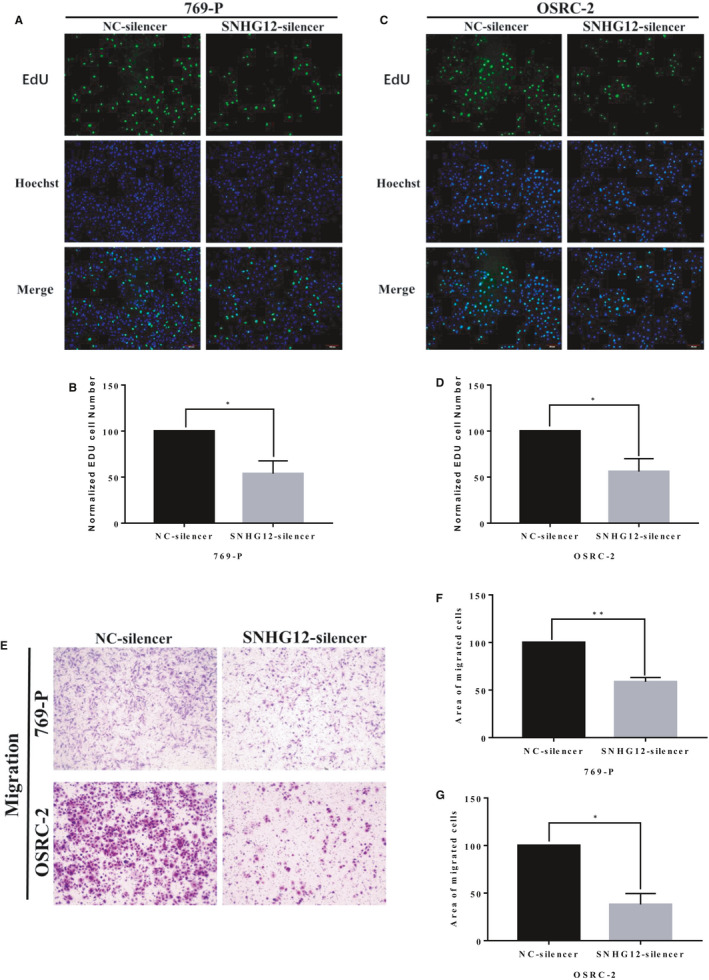
SNHG12 knockdown significantly inhibited proliferation and migration of ccRCC cell lines in vitro. A‐D, EdU staining of ccRCC cell lines, 769‐P and OSRC‐2. SHNG12 silencing significantly inhibited cell proliferation in vitro. E‐G, Transwell assay showed that SNHG12 silencing significantly inhibited cell migration in vitro (X = 200). Cell numbers were counted in 3 random visual fields. Original magnification = 200×, *P < .05; **P < .01

We then analysed the biological functions of SNHG12 in ccRCC cell lines. Expressions of SNHG12 in 5 commonly used ccRCC cell lines were detected by qRT‐PCR analysis (Figure S1A). We next silenced SNHG12 gene expression in 769‐P and OSRC‐2 cell lines by transfecting the cells with small harpin RNAs (shRNA) against SNHG12. The knockdown efficiencies were confirmed by qRT‐PCR (Figure S1B,C). Cell Counting kit‐8 (CCK‐8) assay and 5‐Ethynyl‐2′‐deoxyuridine (EdU) assay were performed to measure cell proliferation. Transwell assay was performed to measure cell migrative capacity. Results suggested that SNHG12 gene silencing significantly inhibited the proliferation (Figure 2A‐D) and migration of ccRCC cell lines (Figure 2E‐G).

### 3.3 SNHG12 directly interacts with has‐miR‐30a‐3p gene in ccRCC cells

**FIGURE 3:**
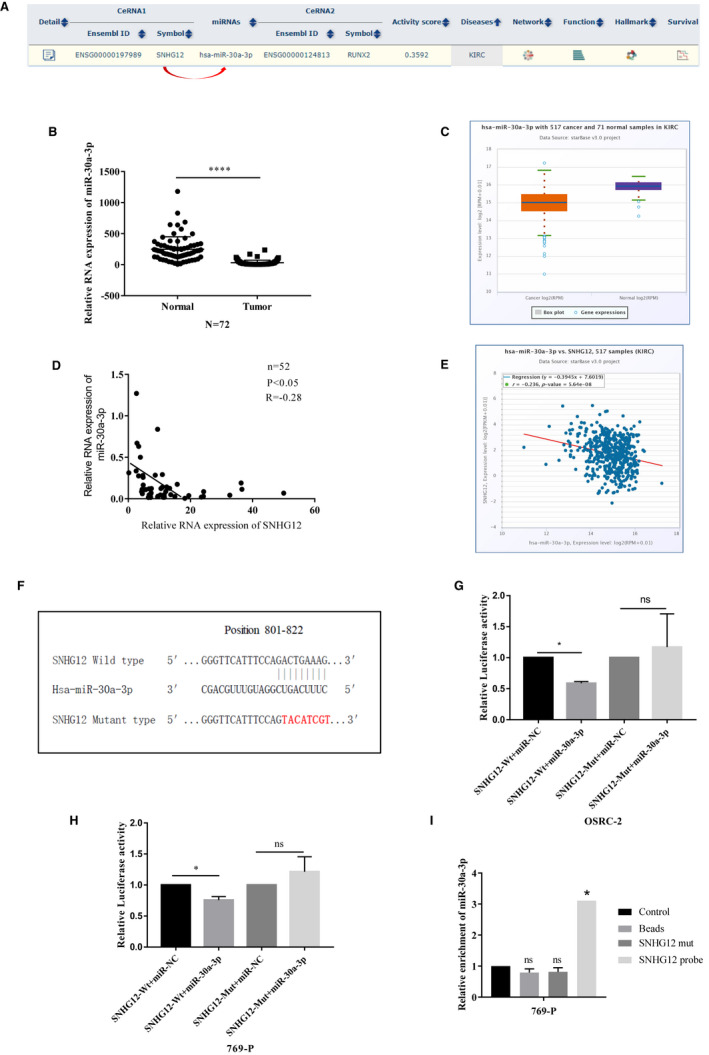
SNHG12 directly interacts with has‐miR‐30a‐3p gene in ccRCC cells. A, Bioinformatic software (ENCORI) suggested that SNHG12 could act as a ceRNA and interacted with has‐miR‐30a‐3p in kidney clear cell carcinoma data set. B, miR‐30a = 3p is significantly down‐regulated in ccRCC tumour tissues compared with adjacent normal tissues. (n = 72, *P* < .001). C, Data from TCGA database suggested that expression of miR‐30a‐3p in ccRCC tumour tissues (n = 523) is much lower than that in normal kidney tissues (n = 100, *P* < .001). D, Two‐tailed Pearson's correlation coefficient test suggested a positive correlation between expression of SNHG12 and miR‐30a‐3p in 52 ccRCC tumour tissues (*P* < .05, *r* = −.28). E, Data from TCGA database suggested a positive correlation between expression of SNHG12 and miR‐30a‐3p in 517 ccRCC tumour tissues (*P* < .01, *r* = −.236). F, Diagram shows the wild‐type and mutant type of miRNA response elements within SNHG12 gene. G and H, Dual luciferase assay suggested that miR‐30a‐3p significantly suppressed the transcriptional level of luciferase vector harbouring wild‐type SNHG12 gene, whereas had no effects on the transcription of luciferase vector harbouring mutant‐type SNHG12 gene (*P* < .05, ns: not significant). I, RNA pull‐down assay was performed to measure the interaction between SNHG12 and miR‐30a‐3p. miR‐30a‐3p was enriched in biotinylated SNHG12 anti‐sense oligo pull‐down product (*P* < .05, ns: not significant)

Our above findings suggested a tumorigenic role of SNHG12 in ccRCC, which is consistent with previous studies. Next, we sought to uncover the mechanisms of SNHG12 involving in the progression of ccRCC. It has been identified that SNHG12 can act as a competitive endogenous RNA (ceRNA) by containing a variety of miRNA binding sites, thereby sponging those miRNAs and regulating the downstream target genes.^28^ Therefore, bioinformatic analysis was performed to analyse the interactions between SNHG12 miRNA response elements (MREs) and cancer‐related miRNAs. With the help of ENCORI software, we noticed that miR‐30a‐3p is very likely to be targeted by SNHG12 (Figure 3A). miR‐30a has been proved to function as a putative tumour suppressor in ccRCC. It inhibits the tumorigenesis of ccRCC by targeting multiple oncogenes such as ZEB2^22^ and WNT2.^25^ We detected the expression of miR‐30a‐3p in the same cohort of ccRCC tumour tissue and adjacent normal renal tissue samples using qRT‐PCR, and the result demonstrated that miR‐30a‐3p was significantly down‐regulated in ccRCC tissue samples (Figure 3B). Data from TCGA database analysed by ENCORI software again confirmed our findings (Figure 3C). We next performed the Spearman's correlation coefficient analysis based on the results from our clinical cohort. We found that expression of SNHG12 was inversely correlated with miR‐30a‐3p expression in ccRCC tissue samples (Figure 3D).

To further validate the interactions between SNHG12 and miR‐30a‐3p, we conducted luciferase reporter plasmids containing the full length of wild‐type SNHG12 gene with or without the predicted MRE sequence mutated (Figure 3E). Next, the reporter plasmids containing wild‐type SNHG12 or mutant‐type SNHG12 were, respectively, co‐transfected with miR‐30a‐3p mimics into two ccRCC cell lines, OSRC‐2 and 769‐p. Dual luciferase reporter assay was performed to measure the transcriptional level of the reporter genes (Figure 3F). Our result revealed that con‐transfection of miR‐30a‐3p significantly decreased the transcription of reporter plasmids harbouring wild‐type SNHG12, however, had no effect on that harbouring mutant‐type SNHG12. Next, we performed an RNA pull‐down assay using a biotin‐labelled SNHG12 anti‐sense probe. Expression of miR‐30a‐3p in control product or probe pull‐down product was measured, and the results suggested that miR‐30a‐3p was significantly enriched in the probe pull‐down product (Figure 3I). This data further confirmed our notion that SNHG12 directly interacts with miR‐30a‐3p gene through its putative miRNA response elements (MREs) in ccRCC cells.

### 3.4 SNHG12 promotes the expression of multiple oncogenes through sponging miR‐30a‐3p

**FIGURE 4:**
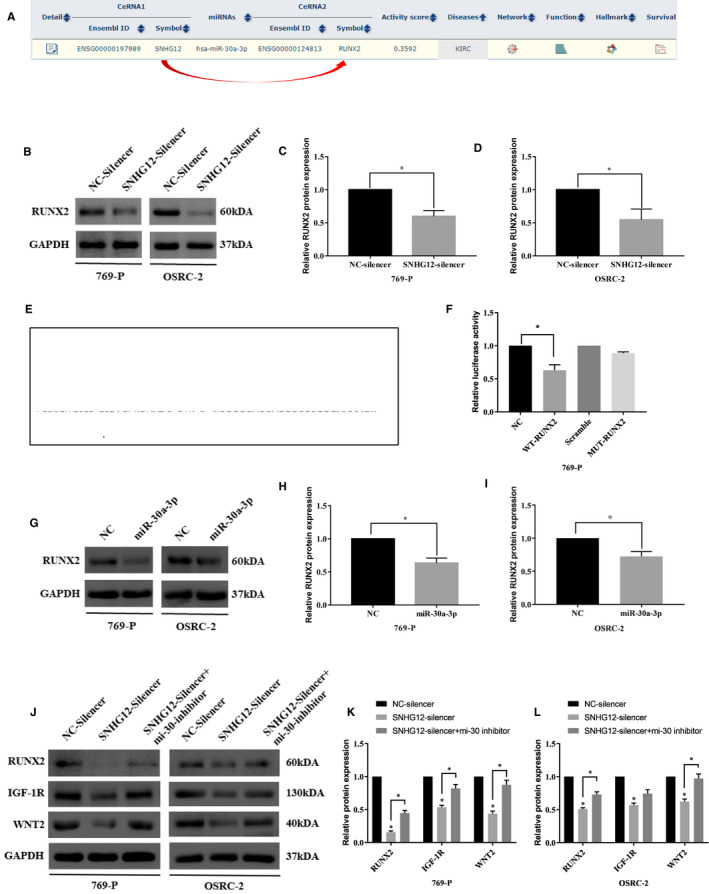
SNHG12 promotes the expression of RUNX2, WNT2 and IGF‐1R via competitively binding with miR‐30a‐3p in ccRCC cells. A, Bioinformatic software (ENCORI) suggested that SNHG12, miR‐30a‐3p and RUNX2 could form a ceRNA network in kidney clear cell carcinoma data set. B‐D, Western blotting analysis suggested SNHG12 knockdown significantly inhibited the expression of RUNX2 in ccRCC cells (*P* < .05). E, Diagram shows the wild‐type and mutant type of miRNA response elements within the 3′UTR region of RUNX2 gene. F, Dual luciferase assay suggested that miR‐30a‐3p significantly suppressed the transcriptional level of luciferase vector harbouring wild‐type RUNX2 gene, whereas had no effects on the transcription of luciferase vector harbouring mutant‐type RUNX2 gene (*P* < .05). G‐I, miR‐30a‐3p overexpression significantly inhibited the expression of RUNX2 in ccRCC cells (*P* < .05). J‐L, Western blotting analysis suggested SNHG12 knockdown significantly inhibited the expression of RUNX2, IGF‐1R and WNT2 in ccRCC cells, whereas silencing of miR‐30a‐3p partially rescued the suppressed expression of RUNX2, IGF‐1R and WNT2 (*P* < .05)

As we identified that SNHG12 competitively interacted with miR‐30a‐3p in ccRCC cells, we then asked which downstream targets may be affected by SNHG12/miR‐30a‐3p axis. Using bioinformatic software,^29^ we located that runt‐related transcription factor 2 (RUNX2) could be a downstream effector and regulated by miR‐30a‐3p mediated ceRNA network (Figure 4A). RUNX2 has been identified to promote cancer cell metastasis by increasing expression of the matrix metalloproteinases and further causing collagen degradation.^30^ A recent study demonstrated that RUNX2 modulates epithelial‐mesenchymal transition (EMT) of ccRCC cells and promotes the aggressiveness of ccRCC.^31^ To verify our hypothesis, we firstly silenced SNHG12 in ccRCC cells and tested the expression of RUNX2 using Western blotting analysis. Result suggested that RUNX2 expression was significantly reduced in response to SNHG12 knockdown (Figure 4B‐D). To further confirm the regulation between miR‐30a‐3p and RUNX2, the miRNA response elements within 3′ UTR region of RUNX2 gene were analysed and two MREs of miR‐30a‐3p were identified by both Targetscan^32^ and miRDB^29^ software (Figure 4E). Luciferase reporter plasmids containing full length of wild‐type RUNX2 or MREs mutated RUNX2 were co‐transfected with miR‐30a‐3p mimics into ccRCC cells, and luciferase assay was performed to examine the transcriptional level of the reporter gene. Result suggested that miR‐30a‐3p significantly reduced the transcription of reported genes contained wild‐type RUNX2 when compared with those carried mutant‐type RUNX2 (4f). Next, the same miR‐30a‐3p mimics were transfected into ccRCC cells, and expression of RUNX2 was examined by Western blotting analysis. In response to miR‐30a‐3p overexpression, RUNX2 expression was significantly repressed (Figure 4G). These results demonstrated that RUNX2 could be a downstream target of miR‐30a‐3p.

We then performed rescue experiment to further confirm the regulatory network. SNHG12 was knocked down alone or knocked down with miR‐30a‐3p silencing. After that, expression of RUNX2, insulin‑like growth factor 1 receptor (IGF‐1R) and WNT2 was examined by Western blot analysis. IGF‐1R^33^ and WNT2^25^ have been reported to be directly regulated by miR‐30a‐3p and act as oncogenes in ccRCC. Our data confirmed that, expressions of RUNX2, IGF‐1R and WNT2 were remarkably down‐regulated in response to SNHG12 knockdown, but could be rescued by miR‐30a‐3p silencing (Figure 4H). The above results suggested that, via the miR‐30a‐3p mediated ceRNA network, SNHG12 could regulate the expressions of multiple downstream genes and stimulated the tumorigenesis of ccRCC.

### 3.5 SNHG12 promotes malignancy phenotypes of ccRCC cells through sponging miR‐30a‐3p in vitro and in vivo

**FIGURE 5:**
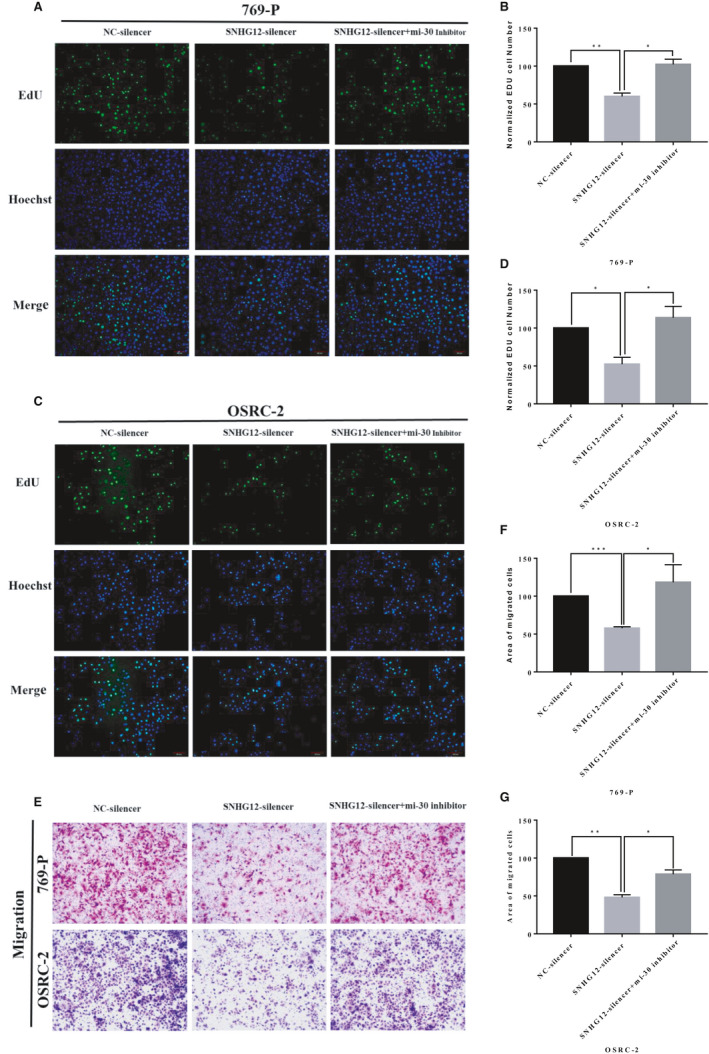
SNHG12 promotes proliferation and migration of ccRCC cells through sponging miR‐30a‐3p in vitro. A‐D, EdU assay suggested that SHNG12 knockdown significantly inhibited cell proliferation, and silencing of miR‐30a‐3p partially rescued the cell growth hampered by SNHG12 knockdown in ccRCC cells. E‐G, Transwell assay showed that SNHG12 knockdown significantly inhibited cell migration, and silencing of miR‐30a‐3p partially rescued the cell migration weakened by SNHG12 knockdown in ccRCC cells. Cell numbers were counted in 3 random visual fields. Original magnification = 200×, *P < .05; **P < .01

To demonstrate that SNHG12 promotes the malignancy phenotypes of ccRCC cells, we then performed rescue experiments. SNHG12 was knocked down in ccRCC cells, and then, the treated cells were subjected to miR‐30a‐3p inhibitor transfection. Cell proliferative and migrative capacities were analysed using EdU assay (Figure 5A,B) and Transwell assay (Figure 5C,D). Results suggested that miR‐30a‐3p knockdown partially rescued the cell proliferation and migration which were significantly hampered by SNHG12 knockdown alone.

**FIGURE 6:**
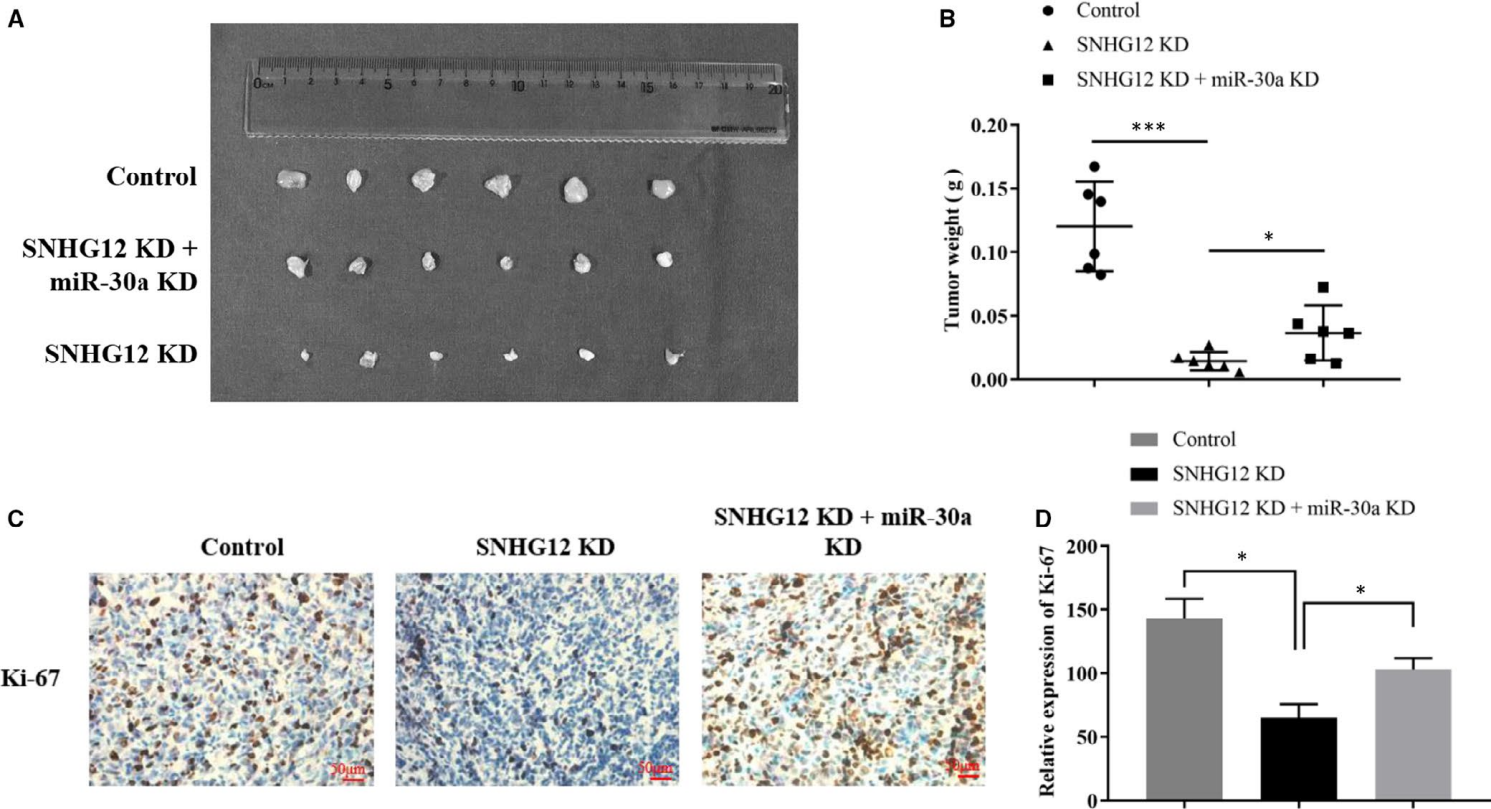
SNHG12 promotes proliferation of ccRCC cells in vivo. A, In vivo tumour lumps of xenograft mouse models composed of 3 types of OSRC‐2 cells: empty vector, SNHG12 knockdown, SNHG12 and miR‐30a‐3p knockdown. B, The mean tumour weight of each group. Data are shown as mean ± SD of the tumour weights, n = 5. *P < .05, **P < .01 (ANOVA). C and D, IHC staining of ki67 in xenograft tumours. The positive‐staining rate was counted and analysed in three random visual fields. *P < .05

Next, we examined the biological function of SNHG12 in vivo using a xenograft model. OSRC‐2 cells were infected with anti‐SNHG12 shRNA to conduct the SNHG12‐knockdown (KD) ccRCC cells. OSRC‐2 cells (Control or SNHG12‐KD cells) were subcutaneously injected into the flanks of nude mice (4‐6 weeks). After xenograft tumours were established, and the mice were separately treated with intratumour injection of miR‐30a‐3p antagomir or vehicle control. The mice were separated into three groups according to treatments: Group 1, mice were injected with control OSRC‐2 cells and followed by intratumour injection of vehicle; Group 2, mice were injected with SNHG12‐KD OSRC‐2 cells and followed by intratumour injection of vehicle; Group 3, mice were injected with SNHG12‐KD OSRC‐2 cells and followed by intratumour injection of miR‐30a‐3p antagomir. The weight of mice and the length of tumours were measured and recorded twice a week. On the 45th day, mice were killed and the size and weight of tumour lumps in each group were measured and compared (Figure 6A and Figure S1D) and subjected to staining assay. Results suggested that SNHG12 knockdown significantly suppressed the xenograft tumour growth, whereas inhibition of miR‐30a‐3p rescued the xenograft tumour growth comparing with the group treated with SNHG12‐KD cells and vehicle control (Figure 6B). Immunochemistry staining assay showed that SNHG12 knockdown effectively suppressed positive‐rate of ki67 in xenograft tumour, whereas miR‐30a silencing partially reversed Ki67 positive‐rate which was significantly suppressed by SNHG12 knockdown (Figure 6C,D).

## 4 DISCUSSION

Accumulating studies have revealed the key regulatory role of lncRNAs in the tumorigenesis of human cancers. lncRNAs exert its ontogenetic or tumour suppressor role through modulating transcriptional or post‐transcriptional expression of downstream cancer‐related factors.^34^, ^35^ SNHG12 has been increasingly recognized to involve in a number of cancers, such as gastric,^36^ glioma^37^ and breast cancers.^14^ Interestingly, SNHG12 has been reported to accelerate the progression of renal cell cancer, and high expression of SNHG12 positively correlates with poor clinical outcome of patients with ccRCC.^9^ Consistently, we have measured the expression of SNHG12 in our ccRCC tissue specimens and confirmed the up‐regulation of SNHG12 in ccRCC tissues. Silencing of SNHG12 significantly inhibits the proliferation and migration of ccRCC cell lines in vitro and in vivo. Bioinformatic analysis suggested that SNHG12 could potentially interact with miR‐30a‐3p gene and interrupt the epigenetic silencing effect on its target genes. Dual luciferase reporter assay was performed to validate the interaction. miR‐30a has been identified to modulate cancer progression via targeting multiple cancer‐related genes. In this study, we discovered the miR‐30a‐miediated crosstalk between SNHG12 and RUNX2, IGF‐1R and WNT2. Mechanistically, SNHG12 stimulates tumorigenesis of ccRCC through the crosstalk with oncogenes via competitively binding with miR‐30a‐3p. Knockdown of miR‐30a partially compromised the malignancy phenotypes of ccRCC cells which was inhibited by silencing of SNHG12 alone.

It is well known that SNHG12 can act as a ceRNA for various miRNAs and modulate the function or expression of their downstream targets. In hepatocellular carcinoma (HCC), SNHG12 interacts with miR‐199a‐5p and consequently up‐regulates the expression of mitogen‐activated protein kinase 3 (MLK3) as well as the phosphorylation of IkB‐α and NF‐kB.^11^ In non‐small cell lung cancer (NSCLC), SNHG12 modulates multidrug resistance via sponging miR‐181a and activating MAPK/Slug pathway.^38^ It has also been demonstrated that in ccRCC, SNHG12 regulates expression of HIF1α via sponging of miR‐199‐5p and increases proliferation of ccRCC cells.^9^ In the present study, we for the first time elucidated that SNHG12 interacts with miR‐30a‐3p, a putative tumour suppressor, and antagonizes its tumour suppressive feature. Noteworthily, via sponging miR‐30a‐3p, SNHG12 increases the expression of RUNX2, IGF‐1R and WNT2 and promotes the proliferative and migrative capacities of ccRCC cells.

miR‐30a precursor gene has been identified as a tumour suppressor in many cancers. Down‐regulation of miR‐30a‐5p is frequently detected in ccRCC tissues, and miR‐30a‐5p is widely demonstrated to facilitate the proliferation, invasion and migration of ccRCC cell lines.^22^ Mechanistically, miR‐30a‐5p inhibits tumour growth and cellular proliferation of ccRCC through targeting glucose‐regulated protein78 (GRP78) and modulating unfolded protein response (UPR) pathway.^23^ miR‐30a‐5p is also reported to suppress ccRCC cell EMT by targeting zinc finger E‐box binding homeobox 2 (ZEB2) mRNA.^22^ miR‐30a‐3p is the other mature miRNA generated from miR‐30a precursor gene and shares a similar tumour suppressor function as miR‐30a‐5p. miR‐30a‐3p is reportedly to be down‐regulated in oesophageal squamous cell carcinoma (ESCC) tissues, and down‐regulation of miR‐30a‐3p promotes cell proliferation of ESCC cells via activating Wnt signalling pathway.^33^ miR‐30a‐3p is also found to play an anti‐tumour role in RCC by targeting WNT2.^25^ Data from our study also confirmed that miR‐30a‐3p suppresses proliferation and migration of RCC cells by inhibiting several target genes, RUNX2, IGF‐1R and WNT2.

In conclusion, our present study revealed the tumorigenic role of SNHG12 in ccRCC, and we also uncovered a novel mechanism that SNHG12 competitively binds with miR‐30a‐3p and up‐regulates the expression of downstream oncogenes, RUNX2, IGF‐1R and WNT2. In vivo experiments also showed that knockdown of SNHG12 significantly inhibited xenograft tumour growth, and co‐silencing of SNHG12 and miR‐30a‐3p partially compromised the tumour growth. Our data provided new evidence of SNHG12 acting as an oncogene in ccRCC and uncovered a new molecular pathway that promotes the progression of ccRCC.

## CONFLICT OF INTEREST

The authors confirm that there are no conflicts of interest.

## AUTHOR CONTRIBUTIONS

**Hongyuan Yu:** Data curation (lead); Formal analysis (lead); Investigation (lead); Resources (lead); Validation (lead); Writing‐original draft (lead). **Junlong Liu:** Data curation (supporting); Resources (supporting). **Zhe Zhang:** Project administration (supporting); Supervision (supporting). **Yuyan Zhu:** Project administration (supporting); Supervision (supporting). **Jianbin Bi:** Funding acquisition (lead); Investigation (supporting); Supervision (supporting). **Chuize Kong:** Funding acquisition (lead); Supervision (supporting).

## ETHICAL APPROVAL

This study has been conducted in accordance with ethical standards and the national and international guidelines. All animal experiments were carried out according to the protocol approved by the China Medical University Guidelines for Use and Care of Animals. This study was approved by the Research Ethics Committee of China Medical University, and all patients signed the written informed consent.

## Supporting information

Fig S1

## Data Availability

The data sets can be provided by corresponding author upon reasonable requests.
