# Supplementary figures and images for: SNHG12 promotes carcinogenesis of human renal cell cancer via functioning as a competing endogenous RNA and sponging miR‐30a‐3p
Source: J Cell Mol Med. 2021 Mar 30;25(10):4696–708. doi: 10.1111/jcmm.16417 (PMC8107103; doi:10.1111/jcmm.16417)

a

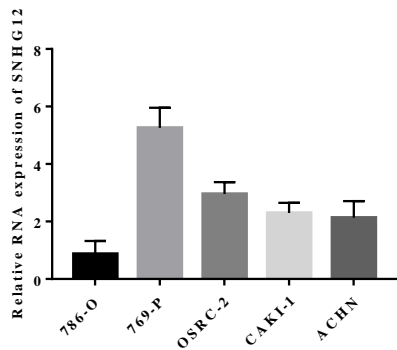

b

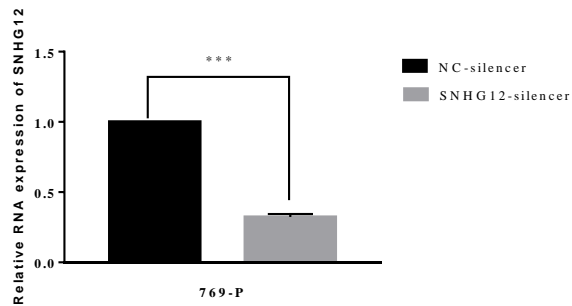

c

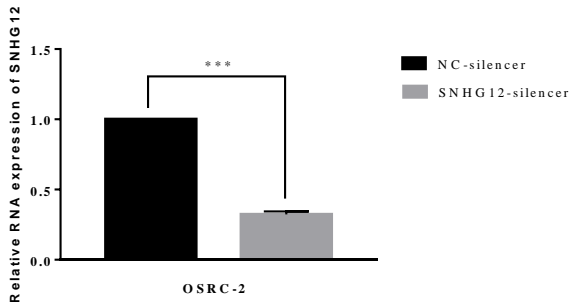

d

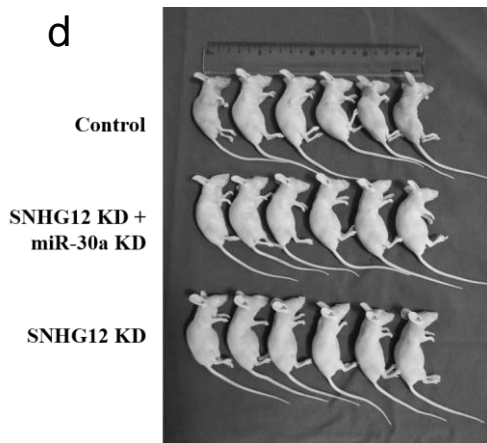

Supplement: Supplementary file 1 — Fig S1 [file JCMM-25-4696-s001.pdf]
